# Low Serum Uric Acid Levels and Risk of Psychiatric Disorders: A Population-Based Cohort Study

**DOI:** 10.1192/j.eurpsy.2026.11798

**Published:** 2026-07-31

**Authors:** R. Leiba, O. Lencovsky, A. Leiba, A. Israel

**Affiliations:** 1 Unicamillus Medical University, Rome, Italy; 2 Nephrology and Hypertension, Assuta Ashdod Hospital, Ashdod; 3 Leumit Research Institute, Leumit Health Services, Tel Aviv, Israel

## Abstract

**Introduction:**

Uric acid (UA) is the end product of purine metabolism and a major plasma antioxidant. While elevated UA is linked to metabolic and vascular risk, low UA may diminish neuroprotection and increase vulnerability to oxidative stress.

**Objectives:**

To assess whether low serum UA levels are associated with a higher incidence of psychiatric and neurocognitive disorders in adults.

**Methods:**

A retrospective cohort study was conducted using electronic health records from Leumit Health Services (HMO) (2002–2024). Adults with ≥1 UA measurement were included. Examinees with elevated uric acid ( >6mg/dl for women, >7mg/dL for men) were excluded. Low UA was defined as <3 mg/dL; others served as controls. Exclusion criteria: malignancy, pregnancy, and insulin use. Any low UA level (even on a single occasion) would commit the examinee to the “low urate” group. Only incident (“New”) diagnoses were analyzed to reflect new-onset psychiatric morbidity during follow-up. Analyses used multivariate logistic regression adjusted for age, sex, socioeconomic status, and comorbidities. Mean follow-up: 3367 ± 578 days (≈9.2 ± 1.6 years).

**Results:**

Among 51,348 participants (25,672 low-UA; 25,676 controls; mean age ≈ 51 years), low UA was associated with significantly higher odds of several psychiatric outcomes (Table 1).

**Table 1.** Incidence and adjusted odds ratios for new psychiatric diagnoses Table 1. Long description.

DIAGNOSIS	LOW UA N (%)	NORMAL UA N (%)	OR (95% CI)	P
DEPRESSION	593 (2.31)	427 (1.66)	1.40 (1.23–1.59)	<0.001
ANXIETY	3,845 (15.0)	3,384 (13.2)	1.16 (1.10–1.22)	<0.001
PTSD	271 (1.06)	214 (0.83)	1.27 (1.06–1.53)	0.009
SCHIZOPHRENIA	175 (0.68)	93 (0.36)	1.89 (1.46–2.46)	<0.001
PERSONALITY DISORDER	292 (1.14)	185 (0.72)	1.59 (1.31–1.92)	<0.001
COGNITIVE DISORDER	170 (0.66)	120 (0.47)	1.42 (1.12–1.81)	0.003
DEMENTIA	234 (0.91)	149 (0.58)	1.58 (1.28–1.95)	<0.001
EPILEPSY	120 (0.47)	60 (0.23)	2.00 (1.46–2.78)	<0.001
HEADACHE	5,013 (19.5)	4,344 (16.9)	1.19 (1.14–1.25)	<0.001

**Conclusions:**

Low serum UA levels are independently associated with an increased risk of depression, anxiety, and other psychiatric and neurocognitive disorders. UA may serve as a biomarker of reduced antioxidant defense and heightened neuropsychiatric vulnerability. Prospective and interventional studies should evaluate whether UA-modulating strategies can aid in mental-health prevention or treatment.

**Disclosure of Interest:**

None Declared

## Long descriptions

### Table 1. Long description

The table contains five columns: DIAGNOSIS, LOW U A N (%), NORMAL U A N (%), O R (95% C I), and P.

* DEPRESSION: Low U A 593 (2.31%); Normal U A 427 (1.66%); O R 1.40 (1.23–1.59); P less than 0.001.

* ANXIETY: Low U A 3,845 (15.0%); Normal U A 3,384 (13.2%); O R 1.16 (1.10–1.22); P less than 0.001.

* P T S D: Low U A 271 (1.06%); Normal U A 214 (0.83%); O R 1.27 (1.06–1.53); P 0.009.

* SCHIZOPHRENIA: Low U A 175 (0.68%); Normal U A 93 (0.36%); O R 1.89 (1.46–2.46); P less than 0.001.

* PERSONALITY DISORDER: Low U A 292 (1.14%); Normal U A 185 (0.72%); O R 1.59 (1.31–1.92); P less than 0.001.

* COGNITIVE DISORDER: Low U A 170 (0.66%); Normal U A 120 (0.47%); O R 1.42 (1.12–1.81); P 0.003.

* DEMENTIA: Low U A 234 (0.91%); Normal U A 149 (0.58%); O R 1.58 (1.28–1.95); P less than 0.001.

* EPILEPSY: Low U A 120 (0.47%); Normal U A 60 (0.23%); O R 2.00 (1.46–2.78); P less than 0.001.

* HEADACHE: Low U A 5,013 (19.5%); Normal U A 4,344 (16.9%); O R 1.19 (1.14–1.25); P less than 0.001.

Navigate back to Table 1..

